# Perinatal Brain Injury As a Consequence of Preterm Birth and Intrauterine Inflammation: Designing Targeted Stem Cell Therapies

**DOI:** 10.3389/fnins.2017.00200

**Published:** 2017-04-10

**Authors:** Madison C. B. Paton, Courtney A. McDonald, Beth J. Allison, Michael C. Fahey, Graham Jenkin, Suzanne L. Miller

**Affiliations:** ^1^Neurodevelopment and Neuroprotection Research Group, The Ritchie Centre, Hudson Institute of Medical Research, Monash UniversityClayton, VIC, Australia; ^2^Department of Obstetrics and Gynaecology, Monash Medical Centre, Monash UniversityClayton, VIC, Australia; ^3^Department of Paediatrics, Monash UniversityClayton, VIC, Australia

**Keywords:** preterm, brain, inflammation, chorioamnionitis, stem cells, endothelial progenitor cells, mesenchymal stem cells, cerebral palsy

## Abstract

Chorioamnionitis is a major cause of preterm birth and brain injury. Bacterial invasion of the chorion and amnion, and/or the placenta, can lead to a fetal inflammatory response, which in turn has significant adverse consequences for the developing fetal brain. Accordingly, there is a strong causal link between chorioamnionitis, preterm brain injury and the pathogenesis of severe postnatal neurological deficits and cerebral palsy. Currently there are no treatments to protect or repair against brain injury in preterm infants born after pregnancy compromised by intrauterine infection. This review describes the injurious cascade of events in the preterm brain in response to a severe fetal inflammatory event. We will highlight specific periods of increased vulnerability, and the potential effects of therapeutic intervention with cell-based therapies. Many clinical trials are underway to investigate the efficacy of stem cells to treat patients with cerebral palsy. Stem cells, obtained from umbilical cord tissue and cord blood, normally discarded after birth, are emerging as a safe and potentially effective therapy. It is not yet known, however, which stem cell type(s) are the most efficacious for administration to preterm infants to treat brain injury-mediated inflammation. Individual stem cell populations found in cord blood and tissue, such as mesenchymal stem cells (MSCs) and endothelial progenitor cells (EPCs), have a number of potential benefits that may specifically target preterm inflammatory-induced brain injury. MSCs have strong immunomodulatory potential, protecting against global and local neuroinflammatory cascades triggered during infection to the fetus. EPCs have angiogenic and vascular reparative qualities that make them ideal for neurovascular repair. A combined therapy using both MSCs and EPCs to target inflammation and promote angiogenesis for re-establishment of vital vessel networks is a treatment concept that warrants further investigation.

## Introduction

Nearly 10% of all births are preterm, <37 weeks completed gestation (Beck et al., 2010). The survival of very preterm infants (28–32 weeks gestation) and extremely preterm infants (<28 weeks gestation) has improved over the past several decades (Miyazaki et al., 2007; Tita and Andrews, 2010), however prematurity still accounts for up to 70% of perinatal deaths and, in survivors, adverse neurodevelopmental outcomes, and cerebral palsy (CP; Singh et al., 2011). After birth there are no clinical treatments to protect or repair the brain of preterm infants. Moreover, whilst survival rates for very/extremely preterm infants have improved, a recent review revealed that the rate of CP in this population remains static (Oskoui et al., 2013). It is known that 40–70% of cases of preterm birth are complicated by inflammation affecting the placenta and its membranes, termed *chorioamnionitis* (Tita and Andrews, 2010). Chorioamnionitis and preterm birth are strongly linked, with rates of chorioamnionitis inversely correlated to fetal gestational age and preterm birth (Galinsky et al., 2013). Chorioamnionitis is implicated in 66% of preterm births at 24 weeks or earlier, with this figure decreasing to 16% by 34 weeks gestation (Lahra and Jeffery, 2004). Independently, both preterm birth and chorioamnionitis contribute to brain injury in infants born preterm and the development of subsequent neurological deficits. However, when these occur simultaneously, the degree of brain injury is more severe.

Acute inflammation of the feto-placental environment, broadly describing chorioamnionitis, is a principal contributor to premature rupture of membranes (PROM) and spontaneous preterm birth (Romero and Mazor, 1988; Tita and Andrews, 2010). PROM does not have to be present for a diagnosis of chorioamnionitis—more than one third of patients that deliver preterm will have intact placental membranes—yet 13% of these patients will later be diagnosed as having chorioamnionitis (Goncalves et al., 2002). Nevertheless, PROM is the most easily identifiable factor for indicating chorioamnionitis, which may be accompanied by vaginal bacterial abnormalities, meconium stained amniotic fluid, prolonged labor and altered placental histopathology (Tita and Andrews, 2010).

Chorioamnionitis causes fetal inflammation and injury to the immature brain, increasing the likelihood of intraventricular hemorrhage (IVH) and diffuse white matter injury (Wu and Colford, 2000). In addition to the fetal inflammatory response, hypoxia also contributes to perinatal brain injury associated with chorioamnionitis and preterm birth (Khwaja and Volpe, 2008). Both fetal inflammation and hypoxia mediate neuropathology, acting to induce breakdown of the blood brain barrier (BBB), neuroinflammation, and oligodendrocyte cell damage (Malaeb and Dammann, 2009). With no specific treatments available to protect the brain injury caused by infection, most cases involving ruptured membranes require the infant to be delivered early to reduce the risk of further compromise. However, by the time of delivery, the infant has been exposed to inflammatory mediators that pose significant risks to the developing brain. The current standard of care following diagnosis of chorioamnionitis during pregnancy is focused on timely delivery of the infant. Accordingly, fetuses that have been exposed to chorioamnionitis are often further compromised by preterm birth (Guzick and Winn, 1985), and the subsequent risks for the immature brain. In particular, chorioamnionitis and preterm birth both have profound adverse effects on the developing white matter of the brain, which is the key etiology in the pathogenesis of CP (Kaukola et al., 2006).

## Chorioamnionitis

Chorioamnionitis is characterized as intra-amniotic infection where bacterial invasion results in acute inflammation of the placenta and/or fetal membranes (Tita and Andrews, 2010). The bacterial species *Mycoplasma* is the most common bacterial form present in cases of chorioamnionitis, however multiple microorganisms may be involved (Romero and Mazor, 1988; Czikk et al., 2011). These include *Streptococcus hominis, Fusobacterium, Gardnerella vaginalis*, and *Escherichia coli*. Up to 25% of all preterm births <37 weeks gestation are complicated by chorioamnionitis (Goncalves et al., 2002). In some cases, chorioamnionitis is diagnosed before labor through maternal symptoms (referred to as *clinical chorioamnionitis*), and may be confirmed after delivery by histologic analysis of the placenta. Most cases are termed *subclinical chorioamnionitis*, with no obvious symptoms during pregnancy, but placental histopathology after preterm birth revealing significant levels of activated neutrophils and macrophages, indicative of infection (Miyazaki et al., 2007; Gisslen et al., 2016).

Neonates who are born with clinical chorioamnionitis may show symptoms of infection, including increased heart rate, decreased blood pressure and impaired cardiac output (Yanowitz et al., 2002). However, subclinical and undiagnosed infection is also implicated in serious adverse perinatal outcomes (Gibbs, 2001). One population-based study involving >250,000 Australian births found an overall incidence of subclinical histologic chorioamnionitis of 22.6%, however less than half of these cases showed a fetal inflammatory response as evidenced by the presence of infection in the umbilical cord and/or chorion (Gordon et al., 2011). The presence of a fetal inflammatory response was strongly associated with spontaneous preterm labor, but chorioamnionitis in the absence of a fetal inflammatory response was correlated to unexplained fetal death (Gordon et al., 2011). These data demonstrate that chorioamnionitis is a significant contributor to stillbirth and adverse neonatal outcomes, often mediated by fetal inflammatory response syndrome.

### Fetal inflammatory response syndrome (FIRS)

FIRS describes inflammation of multiple fetal organs *in utero* as a result of systemic immune activation, and is associated with increased preterm perinatal morbidity and mortality (Romero and Mazor, 1988; Bashiri et al., 2006). FIRS is defined by elevated interleukin-6 (IL-6) fetal plasma concentration in the presence of chorioamnionitis, PROM or preterm delivery (Madsen-Bouterse et al., 2010). The adverse consequences of fetal exposure to inflammation can be significant for the developing brain. There is a strong association between elevated cord blood IL-6 and the risk of CP for the preterm neonate (Yoon et al., 1995).

FIRS is present in the majority of fetuses exposed to chorioamnionitis (Gomez et al., 1997) principally because the fetus is in contact with an infectious amniotic fluid and membrane environment. Pro-inflammatory cytokines, such as IL-6, may also be present in the uteroplacental circulation (Yoon et al., 2000; Andrews et al., 2006). Where chorioamnionitis is present, the fetal lungs, skin, and gut have been shown to initiate a local inflammatory response, which in turn can evolve into a systemic response and FIRS (Kallapur et al., 2014). In some cases, however, FIRS may also be stimulated by blood-borne maternal or placental microorganisms (Tita and Andrews, 2010). Whatever the primary source, FIRS, and in particular elevated fetal circulating IL-6 concentration, is closely associated with preterm birth and adverse neonatal outcomes (Hofer et al., 2013). Specifically, high levels of fetal IL-6 are linked to fetal cardiovascular dysfunctions, and lung and brain pathologies (Galinsky et al., 2013).

### Brain injury

**Figure 1:**
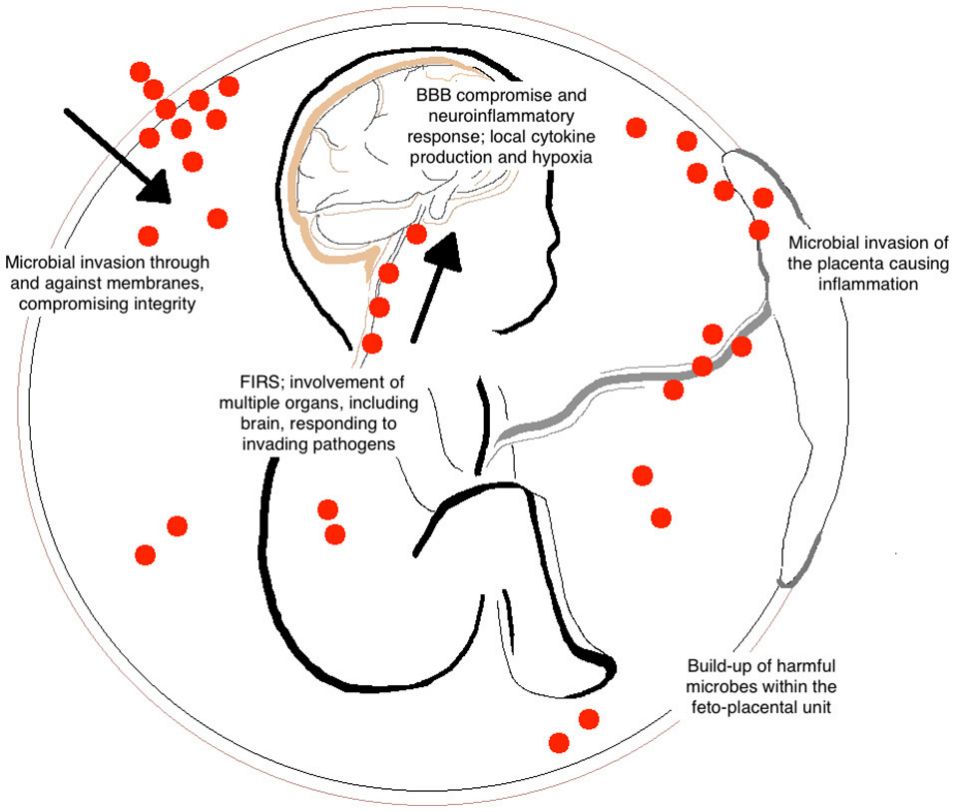
**Contributing factors of brain injury to the fetus following chorioamnionitis**. Bacterial entry during fetal development can occur by many different routes including the placental circulation and through placental membranes (indicated by the red dots). The invasion of microorganisms results in inflammation of the feto-placental unit and the development of fetal inflammatory response syndrome (FIRS). Within the fetus, this can cause hypoxia, blood vessel damage, and blood brain barrier (BBB) compromise. These common responses lead to long-term alterations in white matter development within the fetal brain.

Chorioamnionitis and FIRS are highly associated with neonatal brain injury and the subsequent diagnosis of CP (Shatrov et al., 2010; Kuypers et al., 2012). This is the result of many contributing factors and downstream inflammatory cascades that occur following microbial invasion of the feto-placental unit (Figure 1). Although primarily harmful, inflammatory cascades also involve the recruitment of immune cells and release of growth factors to areas of damage that stimulate endogenous brain repair and regeneration (Dammann and O'Shea, 2008). However, when brain inflammation becomes prolonged or severe, it can exacerbate damage through further influx of cytokines, chemokines, and other inflammatory mediators released from glial cells (Stoll et al., 2003).

Increased circulating pro-inflammatory cytokines, including IL-6, cross the BBB, increase BBB leakiness, and induce a neuroinflammatory response through the activation of microglia and astrocytes (McAdams and Juul, 2012; Schmidt et al., 2016). Pro-inflammatory cytokines that access the fetal brain then act upon vulnerable cell populations, disrupting normal maturation, and development of the white matter in particular (Dammann and Leviton, 1997). Accordingly, the most common neuropathology that occurs in human infants who were exposed to chorioamnionitis is white matter injury, diagnosed by cranial ultrasound, and MRI (Gaudet et al., 2009). The most severe white matter brain injury is generally observed in infants born between 24 and 32 weeks gestation (Volpe, 2009b). Either cystic or diffuse white matter injury bordering the ventricles of the brain, so called periventricular white matter lesions, or periventricular leukomalacia (PVL), is the most common form of brain injury detectable on MRI in patients born preterm and in those with CP (Volpe, 2001; Mann and Horber, 2007). Up to 90% of preterm babies who develop CP will have diffuse or cystic periventricular white matter injury (Krägeloh-Mann and Horber, 2007). It is well-described that this selective vulnerability of the developing white matter of the brain to an inflammatory stimulus is due to cell loss and/or interference with oligodendrocyte maturation (Back et al., 2007). The function of mature oligodendrocytes is to myelinate the developing axons, but impaired myelination is the key pathological feature of PVL. Mature, myelinating oligodendrocytes are present within the periventricular white matter region of the human brain from about 32 weeks gestation but, prior to this, pre-oligodendrocytes predominate (Back et al., 2001). Acute vulnerability of pre-oligodendrocytes is attributed to the predominance of these cells within the white matter (~90% of total oligodendrocytes) between 23 and 32 weeks gestation, with very few mature myelinating oligodendrocytes. If pre-oligodendrocyte development is disrupted, the normal maturation toward the myelinating cells is inhibited. In turn, hypomyelination and disorganization of the white matter is highly correlated with cognitive and functional impairments in children with CP (Hoon et al., 2009).

In addition to PVL, hemorrhage (germinal matrix or intraventricular hemorrhage, GMH/IVH) is a critical contributor to poor neurodevelopmental outcome in preterm infants who were exposed to chorioamnionitis *in utero*. Within the preterm brain, the blood vessels have a relatively low density compared to the term brain, and are easily damaged by inflammatory stimuli which then increases the risk of bleeding and injury to surrounding white matter (Brew et al., 2014). The etiology of hemorrhage within the white matter of preterm infants is multifactorial, but is principally contributed by low vascular density, immaturity of cerebral vessels (with ongoing angiogenesis and vasculogenesis), together with blunted vasoreactivity (Brew et al., 2014). Pathological stimuli, including acute periods of hypoxia-ischemia, and influx of pro-inflammatory cytokines, contributes to brain bleeds with the immature white matter because of the inability of immature vessels to adequately adapt to changing perfusion pressure (Børch et al., 2010). Consequently, brain bleeds occur in up to 50% of infants born preterm at a very low birth weight and can contribute to PVL, with the white matter adjacent to the lateral ventricles becoming coagulated and necrotic (Volpe, 2009a). In contrast, in the very preterm period, the gray matter including cortical regions is highly vascularized and able to withstand injury or mild periods of hypoxia or inflammation (Brew et al., 2014).

Preterm birth and chorioamnionitis is characterized by periods of brain hypoxia, either from inflammation of the placenta disrupting blood flow to the fetus or as a result of inflammation (Baburamani et al., 2012; Stanek, 2013). In response to hypoxia, brain vasculogenesis is upregulated in an attempt to increase brain perfusion, such that capillary networks must sprout and develop to support oxygen and nutrient requirements of the brain (Baburamani et al., 2012). However, these new vessels tend to be more fragile and susceptible to pericytes detachment and cytokine damage. Periods of hypoxia and fluctuations in cerebral oxygenation are exacerbated after birth in preterm infants who require mechanical or assisted ventilation (Barton et al., 2014). This can cause brain damage through oxidative stress and inflammation (Polglase et al., 2012). Therefore, in preterm infants with the additional complication of chorioamnionitis, hypoxia as well as cerebral inflammation causes more severe brain injury (Adams Waldorf and McAdams, 2013).

## Experimental animal models to examine chorioamnionitis

Animal models of chorioamnionitis and fetal inflammation greatly assist the examination of mechanisms underlying neuroinflammation and the progression toward perinatal brain injury. Animal models are critical in assessing the therapeutic potential of various treatments, and lipopolysaccharide (LPS) is one of the most common experimental compounds used to mimic infection. LPS is a component of gram-negative bacterial wall and acts via toll-like receptors (TLRs, specifically TLR 4) to illicit a strong inflammatory response (Briscoe et al., 2006). Experimentally, sterile LPS can be administered during pregnancy into the amniotic cavity or directly to the fetus, to induce a fetal inflammatory response that mimics the inflammatory milieu and white matter brain injury that is observed in human chorioamnionitis (Grigsby et al., 2003; Duncan et al., 2006).

Animal models used to induce chorioamnionitis and a fetal inflammatory response have identified three critical mechanistic components that mediate the progression of perinatal brain injury; breakdown of the BBB, neuroinflammation, and hypoxia, which together contribute to neuropathology (Duncan, 2002; Back and Rivkees, 2004; Schmidt et al., 2016).

### Breakdown of the blood brain barrier (BBB)

The BBB provides the structural interface between circulating blood and cerebral tissue/cerebral extracellular fluid via a cellular barrier composed of endothelial cells (Ballabh et al., 2004). The BBB is a selective barrier, predominantly regulated by gap junctions between endothelial cells, externally lined by perivascular pericytes, and astrocytes, which together form the neurovascular unit (Weiss et al., 2009). In its role of physical separation of the circulatory components from cells within the brain, the BBB maintains brain homeostasis by preventing peripheral toxins and cells from entering the brain, removing waste products from the brain and regulating fluid and metabolic balance (Abbott et al., 2006). Under pathological conditions the BBB may become compromised, and this is shown to be the case in response to a severe fetal inflammatory insult (Ballabh et al., 2004).

Deterioration of the BBB occurs when the tight junctions between endothelial cells are compromised, particularly when membrane proteins are damaged (Nitta et al., 2003; Weiss et al., 2009). BBB compromise can be caused by innate immune activation and by the release of free radicals, prostaglandins, interleukins, glutamate, histamine, and many other substrates (Abbott et al., 2006). Whilst it is not really understood how, or if, endotoxins such as LPS infiltrate the brain, they may elicit damage through direct binding of TLRs on microglia within the brain or via cerebral endothelial cells of the BBB.

### Neuroinflammation

As the brain matures, it is able to counter invading pathogens through innate immunity involving the pattern recognition receptors (PRRs), TLRs (Chen and Nuñez, 2010). However, when activation of these receptors is sustained, BBB permeability is enhanced, and peripheral immune cells gain access to the developing brain (Hagberg et al., 2015). Ongoing TLR activation on immune cells is associated with increased immune cell infiltrates, microglial activation and cytokine release (Cai et al., 2000). It is suggested that, in particular, TLR3 protein expression in the brain is upregulated in cases of white matter injury and invading pathogens in the preterm brain (Vontell et al., 2013). TLR3 is implicated in normal neural cell differentiation and proliferation. When injury to the premature brain occurs, redistribution of TLR3 on microglia over neurons can result in morphological changes in the white matter through the inhibition of axonal growth (Cameron et al., 2007). This is hypothesized to be one of the main reasons that the brain has such limited regenerative capacity following injury.

Microglia are the resident immune cells in the brain that rapidly respond to inflammation or changes in the brain microenvironment (Kreutzberg, 1996). When the BBB becomes compromised, invading blood compounds may activate microglia rapidly; potentially within 60 s (Davalos et al., 2005). Aggregates of activated microglia are indicative of neuroinflammation and ongoing tissue injury through cytokine release (Verney et al., 2012; Adams Waldorf and McAdams, 2013). Microglial activation and cytokine release is also brain region specific; in fetal preterm sheep, LPS injected intra-amniotically increases IL-1β in the fetal brain cortex, hippocampus and cerebellar regions within 2 days (Strackx et al., 2015). After 7 days, microglial and astrocyte proliferation in damaged regions was evident with apoptosis and reduced myelination occurring in the subcortical white matter and hippocampus.

The key cytokine IL-6, as well IL-1α, IL-1β, and IL-18 are released from activated immune cells such as microglia, mast cells, and neutrophils, which in turn stimulate the release of reactive oxygen species (ROS), TNF, and excitatory amino acid agonists including glutamate, that act together to initiate neural cell apoptosis (Bona et al., 1999; Hagberg et al., 2015). These substances can have direct effects on vulnerable oligodendrocyte progenitor cells (OPCs). Three changes occur to OPCs that directly affect white matter development in the premature brain following inflammation: depletion of actively proliferating OPCs, maturational arrest of OPCs, and death of OPCs (Billiards et al., 2008; Volpe et al., 2011). Pro-inflammatory cytokine release and binding of IL1-β and TNF-α to their respective receptors TNF-R_1_ and IL-1R_1_ situated on oligodendrocytes is a principal cause of damage to these cells (Deng et al., 2014). It is known that periods of hypoxia lead to upregulation of TNF-R1 and IL-1 R_1_ in the brain. Neighbouring reactive astrocytes release IL1-β and TNF-α, with signaling through their receptors causing downstream activation of apoptotic pathways and proliferative inhibition of oligodendrocytes (Deng et al., 2014).

### Hypoxia

Animal models of perinatal compromise have elucidated that a fetal inflammatory response directly causes brain hypoxia-ischemia, and also results in an oxygen-independent inflammatory hypoxia, driven via activation of the hypoxia-inducible factor 1α (HIF-1α) pathway (Huang et al., 1998; Duncan, 2002; Peebles et al., 2003). Independently, each of these may contribute to the progression of white matter injury. TLR and NF-κB on placental tissues are activated and cytokine release is upregulated within the placenta and membranes (Briscoe et al., 2006). Pro-inflammatory cytokines cause physical and functional changes in placental tissues, with arteries and veins in the umbilical cord and placenta becoming inflamed (Tita and Andrews, 2010). Inflammation of the cord and the placenta can restrict blood flow and affect placental function. This can lead to acute or prolonged periods of hypoxia-ischemia during fetal gestation.

Within the brain, oxygen-independent hypoxia can occur as a consequence of neuroinflammation due to pro-inflammatory cytokines, ROS, and excitotoxic substances that stimulate anaerobic metabolism. At least in part, this is attributable to the release of nitric oxide from astrocytes following neuroinflammation, causing mitochondrial failure (Ferriero et al., 1996). Hypoxia also inhibits the degradation of HIF-1α (Huang et al., 1998) which may have duel effects to promote repair but also to induce injury (Shi, 2009). For example, HIF-1α regulates genes that mediate cell proliferation and angiogenesis; each of these processes are essential to tissue repair following insult, but aberrant cell proliferation is associated with maturational arrest of oligodendrocytes, and immature angiogenic blood vessels are associated with hemorrhage (Segovia et al., 2008; Brew et al., 2014). Hypoxia is also associated with calcium influx after inflammation-induced glutamate release from immune cells, causing excitotoxicity and resulting in Bax (pro-apoptotic protein) translocation to the mitochondria on OPCs and release of cytochrome-c. These observations indicate that hypoxia has the ability to impair the development of OPCs via activation of cell death through enzymes caspase-9 and caspase-3 (Simonishvili et al., 2013).

It is difficult to tease apart the individual contribution to brain injury that breakdown of the BBB, neuroinflammation, and hypoxia each make. Nonetheless, novel discoveries in large animal models using LPS to stimulate a fetal inflammatory response have revealed that inflammation and hypoxia can independently result in significant white matter injury (Duncan, 2002; Duncan et al., 2006). However, it is most likely that, in preterm infants exposed to chorioamnionitis, both inflammation and hypoxia contribute to the pathogenesis of white matter injury.

There are no approved therapies that can be offered to infants born preterm following exposure to chorioamnionitis, despite our knowledge that these infants are at the greatest risk for brain injury.

## Stem cell therapies

Stem cells have emerged as a promising potential neuroprotective and neuroreparative treatment. Stem cells have the potential for self-renewal as well as the ability to differentiate into mature cell lineages (Bruder et al., 1997; Shen et al., 2004): a feature which allows stem cells to be beneficial for repair, remodeling or new tissue growth after tissue and cell damage. Stem cells also function indirectly to modify endogenous cell responses and can secrete growth factors and cytokines that mediate tissue repair (Baraniak and McDevitt, 2010). Umbilical cord blood (UCB) stem cells, in particular, have a number of benefits that support their therapeutic use for infants after birth who are deemed at high risk for brain injury following antenatal or birth complications. Clinical use of UCB has been well-described since the first UCB cell transplantation in 1988 (M-Reboredo et al., 2000). UCB is a commonly used therapy in a range of hematological conditions like blood cancers as well as graft-vs. host disease. The use of UCB and placental stem cells avoids issues that surround the use of bone marrow derived, and other forms of stem cells that necessitate invasive collection, as the umbilical cord and placenta are normally discarded at birth. Additionally, due to the primitive nature of these cells and a lack of immune recognition surface antigens present on most adult cells, the risk of graft vs. host disease is reduced (Gluckman et al., 1997). Therefore, the risks associated with other more mature types of tissue transplant are minimized.

### UCB clinical trials for CP

Clinical trials have demonstrated the safety of UCB administration to children with established CP (Lee et al., 2012; Feng et al., 2015; Novak et al., 2016). Currently, however, it remains unclear how effective UCB could be in the setting of chorioamnionitis and preterm birth. No trials have been established to assess the efficacy of UCB or stem cell therapies for white matter injuries in preterm newborns. There are however, a number of trials in children and adults that suggest these cells may be an effective therapy.

Novak and colleagues recently reviewed the evidence for the use of stem cells in improving gross motor function in children with CP (Novak et al., 2016); four randomized clinical trials and one non-randomized clinical trial were included for analysis. Two of these trials involved the use of allogeneic UCB transplantation (Min et al., 2013; Kang et al., 2015). All trials reported improvements in motor function following stem cell therapy. However, the two trials using UCB found that treatment, either administered alone or with rehabilitation, resulted in greater motor function improvements than rehabilitation alone. Combining the two studies allowed for the calculation of an odds ratio of 2.62 in favor of UCB therapy for CP. All stem cell trials reported low adverse events (<3%); these included vomiting, breathing difficulties, skin, or eye reactions, with no reported differences in serious adverse events such as hospitalization and seizures. This meta-analysis concluded that stem cell treatment in patients with established CP made a significant short-term improvement in gross motor function after 6 months. We await the results from longer follow-up (>2 years) to ascertain the long-term benefit of stem cell treatment.

Information regarding benefits or risks associated with allogeneic UCB transplantation are pivotal to understanding the types of cell therapy that may be suitable for preterm newborns. In the case of treating those vulnerable to preterm brain injury following chorioamnionitis, allogeneic cell treatment may be the most appropriate. Preterm babies are not the best candidates for autologous UCB transplants for a number of reasons; (i) collection volumes of UCB are proportional to gestational age and preterm birth is therefore implicated in low collection volumes (Wen et al., 2012; Mazzoccoli et al., 2016), (ii) it is unknown how maternal and fetal complications, including gestational diabetes, intrauterine growth restriction, preeclampsia, and chorioamnionitis, predominant in preterm births, alter the relative proportion of stem and immune cells (Wen et al., 2012; Li et al., 2014) and (iii) it has been suggested that, in large clinical trials, such as the Duke University UCB trial (clinicaltrails.gov), recruitment of participants was significantly lower than anticipated (63 recruited vs. a planned recruitment of 120) due to the lack of autologous cord blood storage in children that go on to be diagnosed with CP (Duke Translational Medicine Institute Website, 2012).

## Designing targeted stem cell therapies: MSCs and EPCs

As described above, a main injurious cascade associated with chorioamnionitis and fetal brain injury is inflammation. The major mechanism of action of UCB cells is its anti-inflammatory capacity. This is attributable to the many immunomodulatory cells present in UCB, including mesenchymal stem cells (MSCs; Liao et al., 2013; Zhao et al., 2016). A further injurious cascade related to chorioamnionitis is hypoxia and blood vessel damage. A potent and well-described population of cells in UCB, namely endothelial progenitor cells (EPCs), function to promote neovascularization and vessel repair (Kalka et al., 2000; Murohara et al., 2000). Therefore, a targeted cell therapy, using the cell properties of EPCs and MSCs may be favorable for preterm infants exposed to chorioamnionitis *in utero* (Broxmeyer, 2005; Mei et al., 2010).

### MSCs

MSCs are readily sourced from placental tissues, including the umbilical cord, as well as adult bone marrow and adipose tissue (Hass et al., 2011). The neuroprotective and reparative capacity of MSCs is primarily facilitated by their anti-inflammatory properties and their capacity to secrete neurotrophic and anti-apoptotic factors (Mahmood et al., 2004; van Velthoven et al., 2010). MSCs are well-tolerated due to their low expression of cell surface molecules responsible for the initiation of immune responses, such as low expression of human MHC class 1 and their lack of MHC class 2 molecules (Jacobs et al., 2013).

MSCs prevent inflammatory and apoptotic processes involved in brain injury by actively down-regulating pro-inflammatory cytokines (Ahn et al., 2013; MacFarlane et al., 2013). This was shown in a rodent study where intraventricular hemorrhage was induced immediately following birth and UCB-derived MSCs were transplanted into the brain ventricle (Ahn et al., 2013). Treatment with MSCs suppressed the upregulation of cytokines IL-1α, IL-1β, IL-6, and TNF-α within the CSF and periventricular brain region. This resulted in an attenuation of swelling and fluid formation around the brain and improved behavioral outcomes. The role of MSCs in altering a systemic immune response has been directly demonstrated in an experiment on mice with sepsis (Mei et al., 2010). Six hours after induced sepsis, mice were treated with MSCs derived from bone marrow or saline (control). Results showed that mice treated with MSCs had reduced overall levels of circulating cytokines, down-regulation of inflammatory mediators like IL-6, IL-1β and an up-regulation of pathways responsible for bacterial elimination like C-C chemokine ligand 5 and Fc receptor-mediated immune-cell homing and phagocytosis in macrophages and monocytes. This study supports the use of MSCs for a targeted therapy against infection and inflammation.

MSCs immunomodulatory function is multifaceted, demonstrating not only direct secretion of anti-inflammatory cytokines but also altering immune cell programming and proliferation. MSCs have been shown to modify immune function directly by impairing the differentiation of dendritic cells, the main antigen-presenting cell in human immunity, from monocytes (Ramasamy et al., 2007). MSCs also alter dendritic cell release of pro-inflammatory cytokine TNF-α (Aggarwal and Pittenger, 2005). This can exert long term immune inhibition peripherally, which could be protective in the setting of preterm brain inflammation. T-helper cell production of IFN-γ in the presence of MSCs is also decreased by 50% and IL-4 production increased by over 500% (Aggarwal and Pittenger, 2005). IL-4 release promotes STAT6 pathway activation in MSCs that leads to an increase in TGF-β production (Kyurkchiev et al., 2014). In turn, TGF-β release from MSCs mediates inhibition of inflammatory T-helper cell cytokines, inhibits T-cell proliferation and increases recruitment of T-regulatory cells which help maintain immune tolerance (Zheng et al., 2015). The stimulation of natural killer cells in the presence of MSCs showed a reduced level of IFN-γ secretion by more than 80%. This is important because reducing IFN-γ release from T and natural killer cells inhibits the direct activation of macrophages and stops the activation of pro-inflammatory transcription factors like NF-κβ (Mühl and Pfeilschifter, 2003). These results indicate that MSCs have the capacity to alter macrophage function in the setting of inflammation and brain injury, likely acting via a reduction in immune response.

MSCs also secrete and recruit anti-inflammatory cytokines, growth factors and neurotrophic factors, including hepatocyte growth factor (HGF), brain derived neurotrophic factor (BDNF), vascular endothelial growth factor (VEGF), glial derived neurotrophic factor (GDNF), and IL-10 (Salgado et al., 2015). This is important as exogenous MSCs can promote a more anti-inflammatory environment within the brain, favoring reparative mechanisms. It has been postulated that a key immunomodulatory factor released by MSCs is prostaglandin (PGE2), with one study showing that when PGE2 was inhibited, the immunomodulatory effects of MSCs was abolished (Aggarwal and Pittenger, 2005).

If MSCs are to be used in neonates, expansion of cell populations may be necessary to provide a therapeutic dose. MSC colonies are only found in ~20–30% of all UCB samples collected from term births (Fan et al., 2009), but umbilical cord tissue is a more readily available source of MSCs (Schugar et al., 2009). One gram of umbilical cord tissue can yield up to 11 million cells, of which up to 50% are characterized as mesenchymal progenitors (Schugar et al., 2009). These cells maintain their stem-like phenotype over multiple passages and show high proliferative capability. It has been shown that MSCs passaged up to six times still maintained their stem cell-like phenotype with tri-lineage differentiation potential, maintained expression of typical MSC surface antigens and lack of myeloid or endothelial surface antigens (Ruan et al., 2014). Therefore, a promising source of MSCs for expansion and use therapeutically for preterm brain inflammation is umbilical cord tissue.

If MSCs can be administered at a relevant time point during peak inflammation, they may be able to reduce the pro-inflammatory environment and protect the brain following chorioamnionitis. A mouse model of chorioamnionitis induced via fetal LPS exposure showed that maternal administration of MSCs resulted in reduced IL-6 concentration in the fetal brain (Lei et al., 2015). This was also accompanied by a reduction in activated microglia and improved neurobehavioral outcomes in mice. By suppressing pro-inflammatory mediators and enhancing anti-inflammatory factors, MSCs also create a more supportive environment for repair following brain injury.

Whilst MSCs can be administered following chorioamnionitis to protect against inflammation-induced brain injury, MSCs could potentially be combined with EPCs to support other neuroreparative processes, including vascular repair. Interestingly, MSCs and MSC-like spindle shaped cells have also been shown to support angiogenesis in multiple studies *in-vitro* when co-cultured with EPCs and also *in vivo* in the treatment of experimental hindlimb ischemia (Bhang et al., 2012; Peters et al., 2015).

### EPCs

EPCs can be readily obtained from adult peripheral blood and UCB. EPCs alone may be a promising source for the treatment of inflammation-induced brain injury, with a number of studies reporting that under ischemic conditions, EPCs are able to migrate to regions of tissue damage and/or hypoxia and assist in the neovascularization process (Kalka et al., 2000; Fan et al., 2010).

Endogenous EPCs are well-recognized for their role in vasculogenesis. Vascular remodeling involves the recruitment of stem cells for the formation of new blood vessel and capillary networks, and begins with EPCs becoming mobilized from their primary source, bone marrow (Liu et al., 2007). Mobilization is supported primarily by VEGF, matrix metalloproteinase-9 and erythropoietin (Asahara et al., 1999; Dimmeler, 2010). After this initial phase, endogenous EPCs migrate toward vessel damage by “adhesion rolling” in which the cells attach to surface molecules found on the blood vessel walls (Liu and Velazquez, 2008). VEGF and multiple adhesion molecules including β2 integrins, E-selectin, and P-selectin, assist in this process (Chavakis et al., 2005). EPCs are then able to integrate into the injured endothelial monolayer to support neovascularization and further release of vascular growth factors.

*In vivo* animal experiments support that therapeutically administered EPCs home to regions of tissue damage and participate in vascularization (Kalka et al., 2000; Werner et al., 2003). Neovascularization at sites of damaged brain tissue is vital for cell repair, especially following inflammation and hypoxia. In response to hypoxic injury, blood vessels proliferate and form new networks (Baburamani et al., 2012). This is functionally important for the rapid restoration of oxygen to the brain. However, these newly formed capillaries are unstable and are likely to be dangerous for brain function in the long term, increasing the risk of intracranial hemorrhage (Ballabh, 2010). This abnormal blood vessel development is attributed to the decreased availability of VEGF and reduced EPC mobilization immediately following an insult, and is a phenomenon noted in chorioamnionitis (Kramer et al., 2005; Mooney et al., 2014). Thus, exogenous administration of EPCs in the preterm infant may aid in the establishment of strengthened blood vessel formation that creates more viable and long-lasting vascular networks.

In addition to mediating blood vessel repair, EPCs may have therapeutic benefits through their paracrine activity. EPCs release multiple growth factors and cytokines that contribute to the inhibition of cell death, increased cell proliferation, and recruitment of local stem cells to the site of injury (Tongers et al., 2010). Following an inflammatory or ischemic insult, in which blood flow is impaired, endogenous endothelial cells become activated and initially release nitric oxide, allowing for the expansion of arterioles to compensate for vessel damage. When this expansion is prolonged, blood vessels shunt blood around damaged tissue where vessels have become non-functional and collateral vasculature is formed (Schaper and Scholz, 2003). EPCs themselves are highly receptive to the collateral response whereby they sense stress and endothelial activation, upregulate VEGF receptors and activate Akt signaling which promotes EPC differentiation at sites of damage (Mogi et al., 2008; Tongers et al., 2010). EPCs also release VEGF, stromal cell derived factor (SDF-1), and insulin-like growth factor (IGF-1), which are reparatory soluble factors stimulated following ischemic conditions (Urbich and Dimmeler, 2004; Urbich et al., 2005), with each playing a complimentary role in blood vessel repair. VEGF release is important following brain injury, not only in the formation and repair of blood vessels, but for stimulating axonal outgrowth and the inhibition of hypoxic death of neurons (Sun et al., 2003). VEGF release by EPCs also promotes neurogenesis by direct differentiation and growth of neural precursors. SDF-1 is necessary under inflammatory or ischemic conditions to recruit endothelial progenitors to sites of injury via C-X-C chemokine receptor-4 (CXCR-4; Peplow, 2014). For example, when vessel damage or hypoxia is present in the brain, SDF-1 is released from blood vessels and stem cells, which signals the recruitment of EPCs to the site of injury to aid in repair. IGF-1 is also critical for vascular remodeling and tissue repair as its release into the brain promotes neuronal growth and survival (Madathil et al., 2010). IGF-1 also assists in oligodendrocyte maturation and myelination (Mason et al., 2003). In multiple animal studies of brain ischemia, IGF-1 administration has been shown to reduce neuronal cell death and stimulated glial cell proliferation (Liu et al., 2001; Lin et al., 2009).

These data indicate the significant role that EPCs could play in neuroprotection through the regeneration of strong and long lasting blood vessel networks in brain regions at risk of damage. Thus, while EPCs demonstrate multiple potential neuroprotective functions, it is principally their capacity for blood vessel regeneration that has great use in the setting of chorioamnionitis and neuroinflammation.

Umbilical cord tissue and UCB are excellent sources of MSCs and EPCs, respectively. It is important to elucidate the potential for these cells independently, or as a combined therapy where EPCs and MSCs could be co-administered as a potential targeted therapy for chorioamnionitis and damage to the developing brain.

## Conclusion

Chorioamnionitis and preterm birth are significant contributors to perinatal injury, and often co-exists. The consequences of fetal exposure to infection are complex, mediated by downstream systemic and cerebral hypoxic and inflammatory events that contribute to brain injury. It is known that chorioamnionitis and preterm birth are primary causes of perinatal brain injury and the subsequent development of CP, but currently there are no treatments that could be administered after birth to protect or repair the immature brain. We propose that targeted cell therapy with UCB or cord tissue MSCs and EPCs could be a suitable and efficacious solution. Administration of MSCs soon after birth during the peak phase of neuroinflammation, followed by the subsequent administration of EPCs to induce vascular remodeling, may act to normalize brain development and/or repair the damage consequent to chorioamnionitis. A combined therapy of UCB endothelial progenitor cells and cord tissue MSCs warrants further investigation as a neonatal therapy to reduce the burden of perinatal brain injury and CP.

## Author contributions

All authors contributed to the intellectual data presented in this manuscript, all authors contributed to the preparation of this manuscript, and all authors gave consent to the final submission.

## Funding

This work was made possible with financial support from Inner Wheel Australia, Victorian Government Operational Infrastructure Support Program, a NHMRC Australia Project Grant (APP1081516), ARC Future Fellowship to SM and the Kahli Sargent Research Studentship to MP.

### Conflict of interest statement

The authors declare that the research was conducted in the absence of any commercial or financial relationships that could be construed as a potential conflict of interest.

## Abbreviations

CP: Cerebral palsy
PROM: premature rupture of membranes
IVH: intraventricular hemorrhage
BBB: blood-brain barrier
FIRS: fetal inflammatory response syndrome
IL: interleukin
PVL: periventricular leukomalacia
GMH: germinal matrix hemorrhage
LPS: lipopolysaccharide
TLR: toll-like receptor
ROS: reactive oxygen species
HIF-1α: hypoxia-inducible factor 1α
OPCs: oligodendrocyte progenitor cells
PRR: pattern recognition receptor
HIE: hypoxic-ischemic encephalopathy
ODD: oxygen-dependant domains
UCB: umbilical cord blood
EPCs: endothelial progenitor cells
MSCs: mesenchymal stem cells
MHC: major histocompatibility complex
HE: hepatocyte growth factor
BDNF: brain derived neurotrophic factor
VEGF: vascular endothelial growth factor
GDNF: glial-derived neurotrophic factor
PGE2: prostaglandin
SDF-1: stromal cell-derived factor
IGF-1: insulin-like growth factor
CXCR-4: C-X-C chemokine receptor-4.
